# Supplementary material for: Landscape Homogeneity May Drive the Distribution of Koala Vehicle Collisions on a Major Highway in the Clarke-Connors Range in Central Queensland, Australia
Source: Animals (Basel). 2024 Oct 8;14(19):2902. doi: 10.3390/ani14192902 (PMC11475133; doi:10.3390/ani14192902)
Supplement: Supplementary file 1 [file animals-14-02902-s001.zip › animals-3180874-supplementary.pdf]

Supplementary Materials

Table S1: Regional Ecosystem – Koala Habitat ranking

Regional Ecosystem	Percentage Regional Ecosystem	Koala Habitat rank
11.12.4	100	0
11.3.1	100	0
11.3.11	100	0
11.3.21	100	0
11.4.1	100	0
11.4.4	100	0
11.4.9	100	0
11.8.11	100	0
11.8.13	100	0
11.8.3	100	0
11.9.1	100	0
11.9.1/11.3.1	80/20	0
11.9.1/11.9.5	60/40	0
11.9.4a	100	0
11.9.5	100	0
8.1.1	100	0
8.1.1/8.1.2	90/10	0
8.1.1/8.1.2/8.1.5	80/10/10	0
8.1.1/8.1.3/8.1.2	65/20/15	0
8.1.1/8.1.4/8.1.5	90/5/5	0
8.1.1/8.1.5	70/30	0
8.1.2	100	0
8.1.2/8.1.1	80/20	0
8.1.2/8.1.3	70/30	0

8.1.2/8.1.3/8.1.5	90/5/5	0
8.1.3	100	0
8.1.3/8.1.1	70/30	0
8.1.3/8.1.2	60/40	0
8.1.3/8.1.2/8.1.5/8.1.1	50/30/10/10	0
8.1.3/8.1.4	90/10	0
8.1.3/8.1.5/8.1.1	80/10/10	0
8.1.4	100	0
8.1.4/8.1.5	90/10	0
8.1.5	100	0
8.1.5/8.1.1	60/40	0
8.1.5/8.1.1/8.1.3	50/40/10	0
8.1.5/8.1.1/8.1.4	60/30/10	0
8.11.2	100	0
8.12.10a	100	0
8.12.16	100	0
8.12.1a/8.12.31b/8.12.3a	60/30/10	0
8.12.2	100	0
8.12.2/8.12.17a	70/30	0
8.12.2/8.12.3a	60/40	0
8.12.2/8.12.3a/8.12.19	60/20/20	0
8.12.27a	100	0
8.12.27b	100	0
8.12.31a	100	0
8.12.31b	100	0
8.12.3a	100	0
8.12.3a/8.12.19	80/20	0
8.12.3a/8.12.2	60/40	0
8.12.3a/8.12.2/8.12.19	70/20/10	0
8.12.3b	100	0
8.12.8	100	0
8.2.6a	100	0

8.2.6a/8.2.1	95/5	0
8.2.6a/8.2.11/8.2.1	90/5/5	0
8.3.10/8.3.1a	70/30	0
8.3.11	100	0
8.3.12	100	0
8.3.15	100	0
8.3.1a	100	0
8.3.1a/8.3.10	90/10	0
8.8.1b	100	0
8.1.3/8.1.2/8.3.13a/8.1.1	70/20/5/5	0.05
8.1.3/8.1.2/8.3.13a	60/30/10	0.1
8.1.3/8.1.4/8.3.13a	70/20/10	0.1
8.12.31a/8.12.31b/8.12.5a	70/25/5	0.1
8.3.15/8.3.1a/8.3.3a	50/40/10	0.1
8.3.1a/8.3.3a	90/10	0.1
8.1.3/8.3.13a/8.1.4	70/20/10	0.2
8.12.31a/8.12.5a	90/10	0.2
8.1.3/8.3.13a/8.1.5/8.1.1	60/25/10/5	0.25
11.4.9/11.12.1	90/10	0.3
11.4.9/11.4.2	90/10	0.3
11.12.4/11.12.1a	90/10	0.4
8.3.12/8.1.4/8.3.13c	60/30/10	0.5
11.4.9/11.3.2	80/20	0.6
8.12.10a/8.12.5a/8.12.31a	60/30/10	0.6
8.12.31b/8.12.5a	70/30	0.6
8.3.3a/8.3.1a	60/40	0.6
8.3.13a/8.1.1/8.1.4	70/20/10	0.7
8.3.13a/8.1.4	70/30	0.7
8.3.5/8.3.1a	80/20	0.8
8.3.5/8.3.12/8.3.2	70/15/15	0.85
8.3.5/8.3.3a/8.3.1a	70/15/15	0.85

8.3.5/8.3.2/8.3.11/8.3.3a	75/15/5/5	0.95
11.10.1	100	1
11.8.14	100	1
8.11.3a	100	1
8.3.13a	100	1
8.3.2	100	1
8.3.2/8.3.3a	95/5	1
8.3.3a	100	1
8.3.3a/8.3.5	90/10	1
8.3.5	100	1
8.3.5/8.3.2	80/20	1
8.3.5/8.3.2/8.3.3a	90/5/5	1
8.3.5/8.3.3a	95/5	1
11.3.1/11.3.3/11.3.11	60/30/10	1.2
8.12.5a/8.12.31b	60/40	1.2
8.3.12/8.3.13a/8.3.13c	50/30/20	1.3
11.8.5/11.8.11	70/30	1.4
8.12.23/8.12.7a	60/40	1.4
8.3.3a/8.3.6a	90/10	1.4
8.3.3a/8.3.6a/8.3.5	80/10/10	1.4
11.3.2/11.3.1	50/50	1.5
11.4.9/11.3.4	70/30	1.5
11.8.3/11.8.4	50/50	1.5
11.9.5/11.9.7a/11.9.2	50/30/20	1.5
8.12.12a/8.12.3a	50/50	1.5
8.12.7a/8.12.10a	80/20	1.6
8.3.5/8.3.6a/8.3.3a	80/15/5	1.6
11.9.7/11.9.5	60/40	1.8
8.3.5/8.3.6a	80/20	1.8
11.4.13	100	2
11.8.5	100	2
8.11.4	100	2

8.12.32	100	2
8.12.5a	100	2
8.12.7a	100	2
8.12.7a/8.12.32	60/40	2
11.12.1a/11.12.4	70/30	2.1
11.8.4/11.8.3	70/30	2.1
8.12.7a/8.12.12a	80/20	2.2
8.3.5/8.3.6a/8.3.2	60/30/10	2.2
8.12.5a/8.12.12a	70/30	2.3
8.12.7a/8.12.9	70/30	2.3
11.4.13/11.4.2	60/40	2.4
11.4.2/11.4.9	80/20	2.4
11.5.3/11.7.2	80/20	2.4
8.12.7c/8.12.16	60/40	2.4
11.4.13/11.3.4	80/20	2.6
11.5.3/11.4.13	60/40	2.6
11.9.9/11.11.1	60/40	2.6
8.12.12a/8.12.5a	60/40	2.6
8.12.12a/8.12.7a	60/40	2.6
8.12.12a/8.3.3a	80/20	2.6
11.4.2/11.4.13	70/30	2.7
11.5.3/11.4.9	90/10	2.7
11.4.2/11.4.2/11.4.13	50/30/20	2.8
11.12.1	100	3
11.12.1/11.3.2	90/10	3
11.12.1/11.4.2	70/30	3
11.12.1a	100	3
11.3.10	100	3
11.3.2	100	3
11.4.2	100	3
11.4.2/11.5.2	80/20	3
11.5.2	100	3

11.5.3	100	3
11.5.3/11.3.2	60/40	3
11.5.3/11.4.2	80/20	3
11.5.9c	100	3
11.8.4	100	3
11.9.10	100	3
11.9.2	100	3
11.9.2/11.9.7	60/40	3
11.9.2/11.9.7a	60/40	3
11.9.2/11.9.9	60/40	3
11.9.7	100	3
11.9.7a	100	3
11.9.7a/11.9.9	80/20	3
11.9.7a/11.9.9/11.9.2	50/30/20	3
11.9.9	100	3
11.9.9/11.9.2	80/20	3
8.12.12a	100	3
8.12.4	100	3
8.12.9	100	3
11.12.1a/11.3.25b	95/5	3.05
11.12.1/11.3.25	90/10	3.2
11.12.1/11.3.4	90/10	3.2
11.12.1a/11.3.4	90/10	3.2
11.9.2/11.3.4	80/20	3.4
11.9.9/11.3.2/11.3.25	40/40/20	3.4
8.3.6a/8.3.3a	60/40	3.4
8.3.6a/8.3.5/8.3.3a	60/35/5	3.4
11.12.6a/11.12.4	90/10	3.6
11.5.3/11.3.4	70/30	3.6
8.12.14a/8.12.12a	60/40	3.6
8.3.6a/8.3.1a/8.3.3a	70/15/15	3.65
11.12.3	100	4

11.12.3/11.12.1a	90/10	4
11.12.6a	100	4
11.3.4/11.5.8/11.3.25b	60/30/10	4
11.3.4/11.5.8/11.4.2	60/20/20	4
8.11.1	100	4
8.12.7c	100	4
8.3.6a/8.3.1a	80/20	4
11.3.3/11.3.4/11.3.25	60/30/10	4.4
11.3.4/11.3.2	70/30	4.4
11.3.4/11.12.1a/11.3.25b	65/30/5	4.65
11.3.25	100	5
11.3.25b	100	5
11.3.27b	100	5
11.3.27f	100	5
11.3.4	100	5
11.3.4/11.3.25	90/10	5
11.3.4/11.3.27b	70/30	5
8.11.5a	100	5
8.3.13c	100	5
8.3.6a	100	5
